# Lipoxin A4 reverses mesenchymal phenotypes to attenuate invasion and metastasis via the inhibition of autocrine TGF-β1 signaling in pancreatic cancer

**DOI:** 10.1186/s13046-017-0655-5

**Published:** 2017-12-11

**Authors:** Liang Zong, Ke Chen, Zhengdong Jiang, Xin Chen, Liankang Sun, Jiguang Ma, Cancan Zhou, Qinhong Xu, Wanxing Duan, Liang Han, Jianjun Lei, Xuqi Li, Qingyong Ma, Zheng Wang

**Affiliations:** 1 Department of Hepatobiliary Surgery, First Affiliated Hospital, Xi’an Jiaotong University, 277 West Yanta Road, Xi’an, 710061 China; 2 Department of Emergency, Peking Union Medical College Hospital, 1 Shuaifuyuan Wangfujing Dongcheng District, Beijing, 100730 China; 3 Department of Anesthesiology, First Affiliated Hospital, Xi’an Jiaotong University, 277 West Yanta Road, Xi’an, 710061 China; 4 Department of General Surgery, First Affiliated Hospital, Xi’an Jiaotong University, 277 West Yanta Road, Xi’an, 710061 China

**Keywords:** Pancreatic cancer, Lipoxin A4, Mesenchymal phenotypes, TGF-β1, Invasion and metastasis

## Abstract

**Background:**

Pancreatic cancer is a lethal disease in part because of its potential for aggressive invasion and metastasis. Lipoxin A4 (LXA4) is one of the metabolites that is derived from arachidonic acid and that is catalyzed by 15-lipoxygenase (15-LOX), and it has recently been reported to exhibit anti-cancer effects. However, the role of LXA4 in pancreatic cancer remains to be elucidated.

**Methods:**

Pancreatic cell lines were treated with vehicle or LXA4, and the invasive capacity was then assessed by Transwell assays. The expression of epithelial and mesenchymal markers was determined by western blotting and immunofluorescence. Anti-TGF-β1 neutralizing antibody and exogenous recombinant human TGF-β1 (rhTGF-β1) were used to study the effect of LXA4 on the TGF-β signaling. A liver metastasis model was applied to investigate the effect of LXA4 in vivo*.* The correlation between the Lipoxin effect score (LES) and the clinical-pathological features of pancreatic cancer was also analyzed.

**Results:**

We found that in patients with pancreatic cancer, low LES was correlated with aggressive metastatic potential. The LXA4 activity, which was mediated by the LXA4 receptor FPRL1, could significantly suppress invasion capacity and mesenchymal phenotypes. The expression and autocrine signaling pathway activity of TGF-β1 were also downregulated by LXA4. In the liver metastasis model in nude mice, the stable analog of LXA4, BML-111, could inhibit the metastasis of pancreatic cancer cells.

**Conclusion:**

Our results demonstrated that LXA4 could reverse mesenchymal phenotypes, which attenuated invasion and metastasis via the inhibition of autocrine TGF-β1 signaling in pancreatic cancer, which may provide a new strategy to prevent the metastasis of pancreatic cancer.

## Background

Pancreatic cancer is a lethal malignancy that is characterized by aggressive invasion and metastasis [1]. Due to the absence of early symptoms, patients are usually diagnosed when local invasion or distant metastasis have already occurred. Although some patients are diagnosed early enough so that they can receive surgery, the high rate of recurrence and delayed metastasis are still issues that remain to be resolved. Thus, it is urgent that effective strategies that attenuate the invasion and metastasis of pancreatic cancer be established.

Invasion and distant metastasis have been regarded as hallmarks of cancer [2]. Several mechanisms have been reported to regulate the invasion and metastasis of pancreatic cancer, including epithelial-mesenchymal transition (EMT). EMT is a metastasis-promoting process in which cancer cells lose epithelial phenotypes such as E-cadherin expression and acquire mesenchymal characteristics including elevated expression of N-cadherin and Vimentin expression. A set of transcription factors, such as Snail, Slug and Twist, regulate EMT and cell invasion. Cancer cells with mesenchymal phenotypes exhibit plasticity, which facilitates the deformation, migration and, consequently, metastasis. Our previous studies have also suggested that the induction of EMT significantly promoted cell invasion in both pancreatic cancer [3] and hepatocellular carcinoma [4].

Transforming growth factor-β (TGF-β) is a well-known chemokine that plays an important role in cancer progression and contributes to disease deterioration [5]. The functions of TGF-β in pancreatic cancer include the promotion of proliferation, the inhibition of apoptosis, and the induction of fibrosis [6]. Moreover, the TGF-β pathway is frequently genetically altered in pancreatic cancer. Notably, TGF-β is a key activator of EMT and it induces EMT through either the canonical TGF-β/Smad pathway or non-canonical pathways, such as the MEK/ERK and PI3K/Akt pathway [7, 8]. We have also efficiently induced EMT by exogenous TGF-β1 [9].

Lipoxins, specifically lipoxin A4 (LXA4), are metabolites that are derived from endogenous arachidonic acid and that are catalyzed by 15-lipoxygenase. After combination with a G-protein coupled receptor named formyl peptide receptor like-1 (FPRL1), LXA4 exhibits powerful anti-inflammatory and pro-resolution functions [10]. In the past few decades, the idea that LXA4 tends to attenuate cancer progression has been widely noted [11, 12]. We have previously revealed that LXA4 inhibited cell invasion in pancreatic cancer [13]. However, the mechanisms by which LXA4 attenuates invasion and metastasis have yet to be clarified.

In the present study, we demonstrated that LXA4 could not only reverse the EMT but also inhibit the invasion and metastasis in vitro and in vivo in pancreatic cancer. In addition, the effects of LXA4 could be mediated in part by suppression of the autocrine TGF-β1 signaling.

## Methods

### Materials

5(S), 6(R)-Lipoxin A4 (LXA4) and 5(S), 6(R)-7-trihydroxymethyl Heptanoate (BML-111) were purchased from Cayman Chemical (Ann Arbor, MI, USA). Antibodies against E-cadherin, N-cadherin, Vimentin, and Snail were obtained from Cell Signaling Technology (Boston, MA, USA). The antibodies against FPRL1 and 15-LOX were purchased from Abcam (Cambridge, UK). The anti-TGF-β1 neutralizing antibody was obtained from R&D Systems (Minneapolis, MN, USA), and recombinant human TGF-β1 (rhTGF-β1) was obtained from PeproTech (Rocky Hill, NJ, USA). Anti-β-actin was purchased from Sigma-Aldrich (St. Louis, MO, USA).

### Cell culture

The human pancreatic cancer cell lines Panc-1 and MIA PaCa-2 were purchased from the Cell Bank of the Chinese Academy of Sciences (Shanghai, China). Both of these cell lines were cultured in Dulbecco’s Modified Eagle’s Medium (DMEM) (Gibco, Grand Island, NY, USA) supplemented with 10% fetal bovine serum (FBS) (ExCell, South America), 100 U/mL penicillin and 100 μg/mL streptomycin (Gibco). The cells were cultured at 37 °C in an atmosphere of 5% CO2.

### Cell invasion assay

Matrigel invasion assays were performed to examine the invasive capacity of cancer cells. The upper surface of the membrane was coated with Matrigel (BD Biosciences, Franklin Lakes, USA). Cancer cells (5 × 10^4^) were suspended in FBS-free media then seeded in the upper chamber. In the lower chamber, DMEM supplemented with 10% FBS was added to generate a gradient to drive cell migration. Forty-eight hours later, the medium was aspirated and noninvasive cells in the upper chamber were removed by a cotton swab. The invading cells were then fixed in 4% paraformaldehyde and stained by crystal violet; the number of cells on the membrane in 10 random fields was counted under a microscope. The values reported here are the averages of triplicate experiments.

### Wound-healing assay

Wound-healing assays were performed to evaluate the migration ability of cancer cells. Pancreatic cancer cells were seeded in fibronectin-coated 6-well plates. After the cells reached the appropriate confluence, they were starved in media with 1% FBS overnight, and then, the monolayers were scratched with a 10-μL pipette tip. The cancer cells were washed 3 times with PBS and cultured in FBS-free medium. Photographs of the same locations were obtained at 0 and 24 h under a phase-contrast microscope at 200× magnification. The original borders were marked by red lines, and 24 h later, the number of cells between the borders was counted. This assay was repeated three times and the averages were calculated as the final results.

### Western blotting analysis

Panc-1 and MiaPaCa-2 cells were lysed in RIPA lysis buffer with proteinase inhibitor (Roche, Mannheim, Germany) as previously described [13]. Total protein was electrophoresed in a 10% SDS-PAGE gel and transferred to PVDF membranes (Roche). The membranes were blocked for 2 h in TBS containing 0.1% (vol/vol) Tween-20 and 10% (wt/vol) nonfat dry milk powder and then incubated with the primary antibodies overnight at 4 °C. Following the incubation with the secondary HRP-coupled antibodies for 2 h at room temperature, the membranes were washed with 0.1% TBS-Tween-20, and the immunocomplexes were detected using the enhanced chemiluminescence (ECL) kit and a Molecular Imager ChemiDoc XRS System (Bio-Rad Laboratories, Hercules, CA, USA).

### RNA interference

SiRNA targeting FPRL1 (siFPRL1: 5′-CGGUUUGUCAUUGGCUUUATT-3′, 5′-UAAAGCCAAUGACAAACCGTT-3′) and a negative control (siNC: 5′-UUCUCCGAAGGUGUCACGUTT-3′, 5′-ACGUGACACGUUCGGAGAATT-3′) were synthesized by GenePharma (Shanghai, China). Cells (2 × 10^5^) were seeded into 6-well plates and treated with 100 nM siRNA using Lipofectamine™ 2000 (Invitrogen, CA, USA) according to the manufacturer’s instructions. The cells were used for further experiments after 48 h of transfection.

### Immunofluorescence

Cancer cells were washed with phosphate-buffered saline (PBS) and fixed in 4% paraformaldehyde. After permeabilization with 0.5% Triton X-100 and blocking in 5% bovine serum albumin (BSA), the cells were incubated with primary antibodies overnight at 4 °C then incubated with fluorescence-conjugated secondary antibodies at room temperature for 1 h. The nuclei were stained with 4′, 6-diamidino-2-phenylindole (DAPI) for 5 min. Images were pseudocolored using a Zeiss Instruments confocal microscope.

### Enzyme-linked immunosorbent assay (ELISA)

Cells were treated by corresponding agents for 24 h then cultured in FBS-free medium for 72 h. Then, the culture medium was collected and centrifuged at 1500 rpm for 5 min. The supernatant was frozen at −80 °C. TGF-β1 in the supernatant was detected by a commercial ELISA kit (R&D Systems) according to the manufacturer’s instructions.

### Animal experiments and immunohistochemical staining

Sixteen five-week-old female BALB/c nude mice were purchased and maintained in the animal center of the Medical College of Xi’an Jiaotong University, China. All experiments were approved by the Ethical Committee of the First Affiliated Hospital of Xi’an Jiaotong University.

A reliable liver metastasis model was established according to the methods of our previous study [14]. After anesthetization with 4% chloral hydrate (0.1 mL/10 g), a small incision, which was carefully anatomized to expose the spleen, was made in the left abdominal flank of the mice. Next, Panc-1 cells (5 × 10^5^), which were suspended in 20 μL of Ca^2+^- and Mg^2+^-free HBSS were injected slowly into the subsplenic membrane with a 30-gauge needle. The presence of a vesicle in the spleen was the criterion for successful inoculation; any mice in which leakage occurred were excluded from further analysis. The spleen was replaced and the peritoneum and skin were sutured. Finally, the liver metastasis model was successfully established in twelve mice.

The mice were randomly divided into two groups either treated with vehicle or BML-111. The administration schedule is shown in Fig. 5a. At the end of the eighth week, after the body weight was recorded, mice were sacrificed, and the livers and primary splenic tumors were gently removed. The weights of the livers and the visible metastatic lesions of each mouse were measured. Each primary tumor was randomly dissected into 6 pieces, which were observed under a microscope. The relative areas of the metastatic lesions were measured by Image Pro Plus 6.0 software, and the average number of relative metastatic areas per mouse was calculated.

The tumor samples were also fixed in 4% paraformaldehyde and embedded in paraffin. Serial sections of 4 mm were cut for hematoxylin and eosin (H&E) staining and immunohistochemical staining for TGF-β1 and Snail, as previously described [14].

### Collection of human pancreatic cancer samples and histological analysis

Sixty-six paraffin-embedded pancreatic cancer samples from patients with complete medical records were collected from the First Affiliated Hospital of Medical College, Xi’an Jiaotong University. Sixty-two patients were diagnosed with pancreatic ductal adenocarcinoma, and all of the patients had undergone chemotherapy or radiation therapy prior to surgery. The histological grade and TNM status were confirmed by experienced pancreatic pathologists. All of the samples were subjected to immunohistochemical staining for 15-LOX, FPRL1 and TGF-β1. The staining was scored by two independent investigators according to the methods described previously [15, 16] as negative (0), weak (1), moderate (2) or strong (3).

### Statistical analysis

The data are presented as the mean ± standard deviation (SD). Differences between two groups were determined by Student’s *t* test, while differences among more than two groups were analyzed by analysis of variance (ANOVA) with Dunnett’s test for post-hoc analysis. The correlation between the LES and the clinical-pathological features was determined by Pearson χ^2^ test. *P* < 0.05 was considered significant. All statistical analyses were performed using SPSS 18.0 software.

## Results

### LXA4 inhibits the migration and invasion of pancreatic cancer cells

**Fig. 1:**
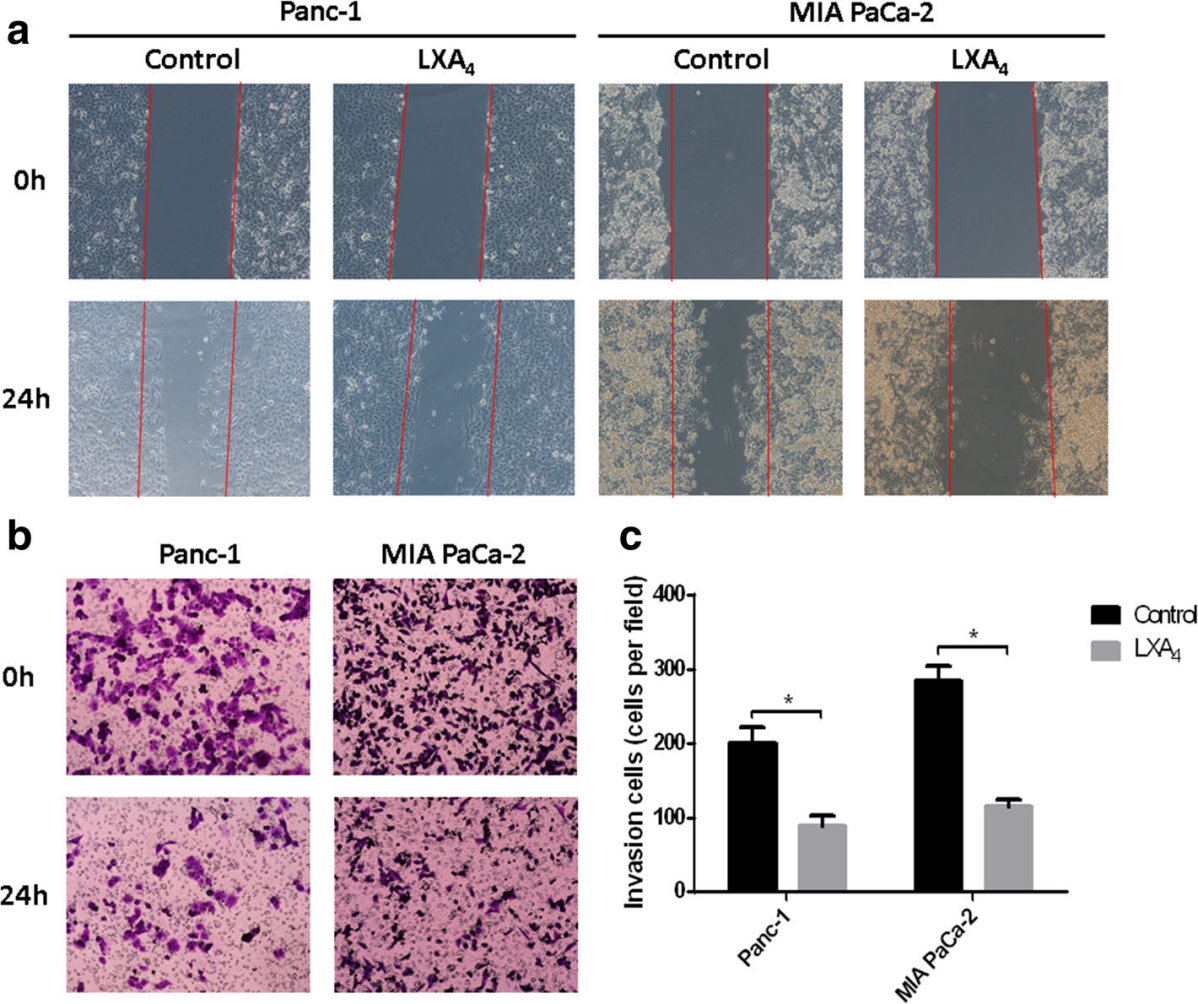
LXA4 inhibits migration and invasion of pancreatic cancer cells. **a** Wound-healing assays were performed to evaluate the migration of Panc-1 and MIA PaCa-2 cells. At 0 hour, cells were treated with either vehicle (methanol) or LXA4 (400 nM), and photographs were taken at 0 and 24th hour. The original borders were marked with red lines. **b** and **c** The two cell lines treated by vehicle or LXA4 were seeded into matrigel-coated chambers to perform transwell assays and the numbers of passed cells were counted in 10 random fields under 200× magnification at 0 and 24th hour. *, *P* < 0.05. All data were obtained from at least three independent experiments

To confirm the inhibitory functions of LXA4 on cell migration and invasion, Panc-1 and MIA PaCa-2 cell lines were treated with either vehicle or LXA4 (400 nM) for 24 h. Then, wound-healing assays were performed (Fig. 1a). We found that, after exposure to LXA4, the migration of both Panc-1 and MIA PaCa-2 cells was significantly suppressed compared to those cells treated with vehicle. To test cell invasion capability, Transwell assays were performed (Fig. 1b, c), and the results showed that cell invasion was significantly attenuated by LXA4 in Panc-1 (*P* = 0.0018) and MIA PaCa-2 (*P* = 0.0002) cells. These data revealed that LXA4 could suppress pancreatic cell migration and invasion.

### LXA4 reverses mesenchymal phenotypes of pancreatic cancer cells via FPRL1

**Fig. 2:**
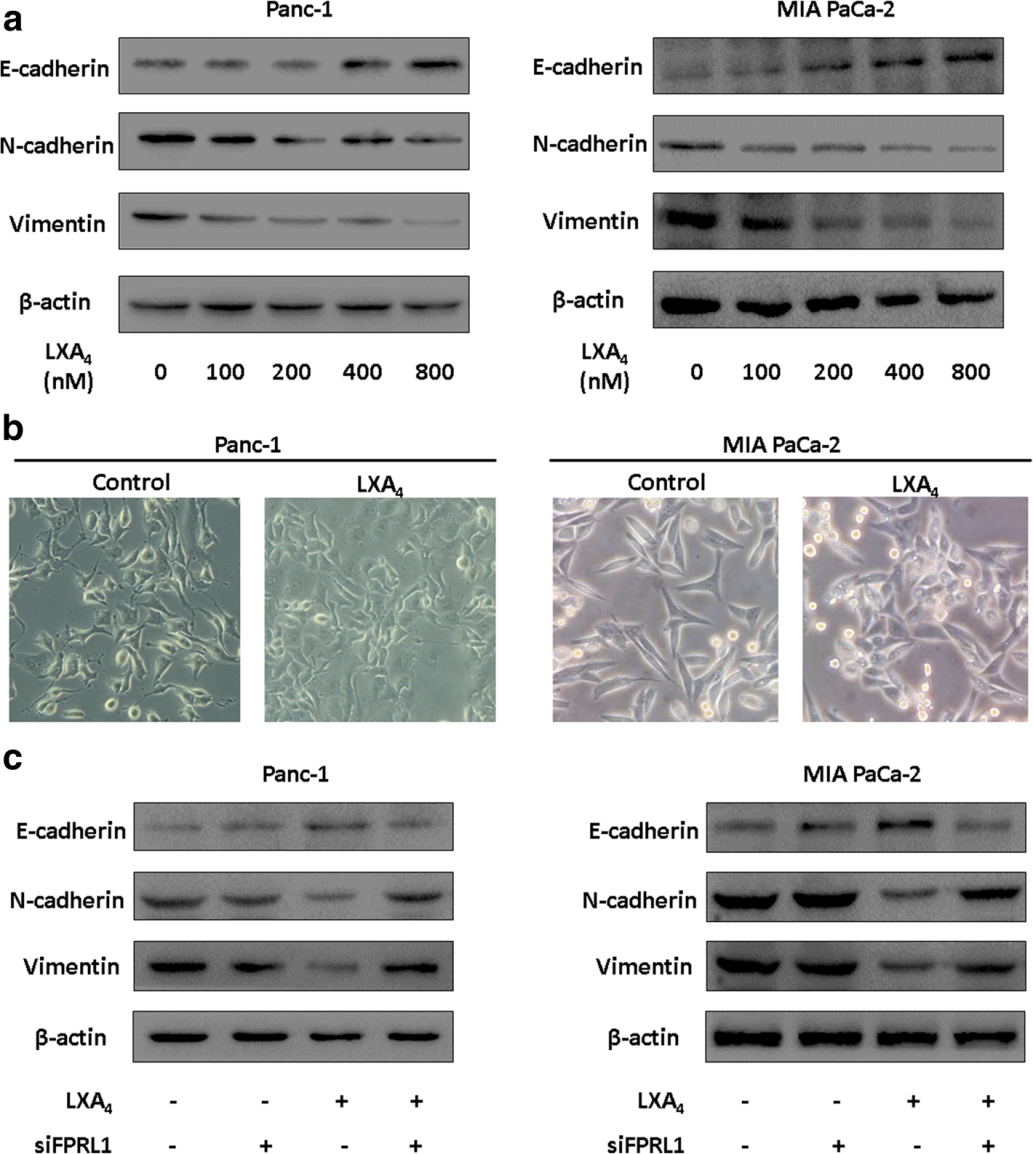
LXA4 reverses mesenchymal phenotypes of pancreatic cancer cells mediated by FPRL1. **a** Panc-1 and MIA PaCa-2 cells were treated with vehicle or gradient concentration of LXA4 (0, 100, 200, 400, 800 nM) for 48 h. Then E-cadherin, N-cadherin and Vimentin were detected by western blotting. **b** The morphology was recorded with or without 400 nM LXA4 treatment for 48 h. **c** Both of the cells were transfected with siRNA targeting FPRL1 (siFPRL1) or negative control (siNC), and 48 h later, 400 nM LXA4 or vehicle were used to treat these cells for 48 h. Then E-cadherin, N-cadherin and Vimentin were detected by western blotting

Next, we set out to investigate the effect of LXA4 on the EMT of pancreatic cancer cells. Gradient concentrations of LXA4 were used to treat Panc-1 and MIA PaCa-2 cells, and then, the expression of E-cadherin, N-cadherin and Vimentin was determined by western blotting. We found that, in parallel with LXA4 concentration, the expression of E-cadherin was gradually up-regulated while the expression of N-cadherin and Vimentin was down-regulated. These data indicated that LXA4 could reverse mesenchymal phenotypes in a dose-dependent manner (Fig. 2a). Based on morphological observations, when these cells were treated with LXA4 (400 nM) for 24 h, both cell lines partially lost their mesenchymal morphology, such as spindle shapes and tight intercellular connections, and became cubic-shaped cells that were linked with one another (Fig. 2b).

As the anti-inflammatory function of LXA4 is mediated by its receptor, FPRL1, we wondered whether FPRL1 plays a role in the maintenance of mesenchymal phenotypes in pancreatic cancer. SiRNA targeting FPRL1 was applied to knock down the expression of FPRL1 in Panc-1 and MIA PaCa-2 cells; the expression of EMT-associated markers was then assessed. In the absence of LXA4, the knock down of FPRL1 expression demonstrated little effect on these markers (Fig. 2c). However, when FPRL1 was knocked down, LXA4 failed to reverse mesenchymal phenotypes in both cell lines (Fig. 2c), which suggested that the effects of LXA4 were mediated by FPRL1.

### LXA4 down-regulates the expression of TGF-β1 via FPRL1

**Fig. 3:**
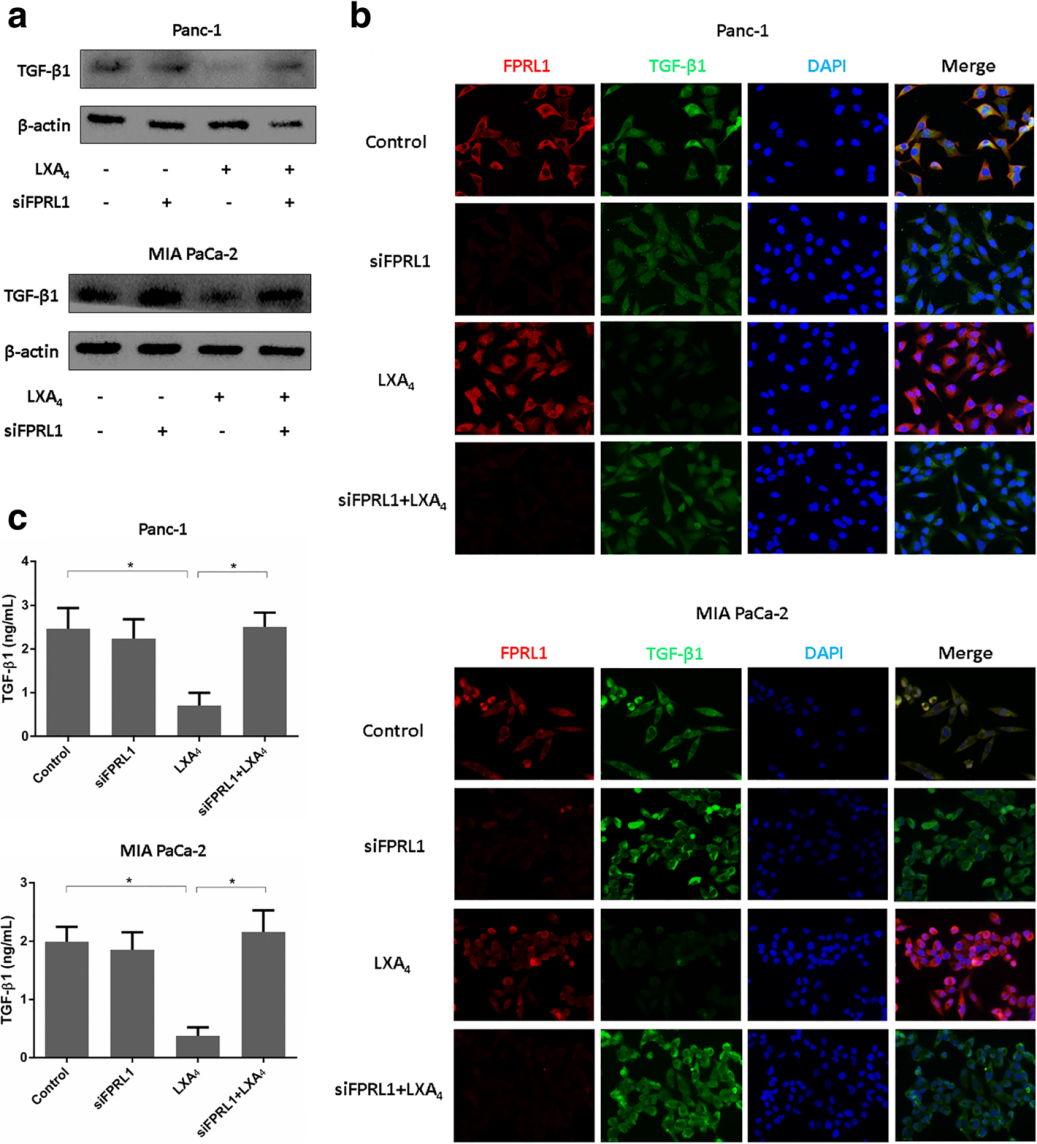
LXA4 downregulates expression of TGF-β1 via FPRL1. **a** Both Panc-1 and MIA PaCa-2 cells were transfected with siRNA targeting FPRL1 (siFPRL1) or negative control (siNC), and 48 h later, 400 nM LXA4 or vehicle were used to treat these cells for 48 h. Then TGF-β1 was detected by western blot. **b** Immunofluorescence was used to detect FPRL1 and TGF-β1 in both cell lines administered as previous description. **c** TGF-β1 was measured by ELISA in the former two cell lines. *, *P* < 0.05. All data were obtained from at least three independent experiments

TGF-β1 is a canonical cytokine that promotes EMT and maintains the mesenchymal phenotype of cancer cells [17].Given the previous evidence that LXA4 can suppress many types of cytokines present during inflammatory responses [18], we inferred that LXA4 might down-regulate the level of TGF-β1. To verify our hypothesis, we determined the expression of TGF-β1 by western blotting. We found that TGF-β1 was down-regulated by LXA4, but when we knocked down FPRL1 by siRNA, LXA4 no longer exerted an effect on TGF-β1. Both Panc-1 and MIA PaCa-2 cells showed a similar result (Fig. 3a). Immunofluorescence was then performed to confirm the results. Treatment of both cell lines with LXA4 decreased the fluorescence intensity of TGF-β1, whereas when FPRL1 expression was knocked down prior to LXA4 administration, TGF-β1 exhibited a similar fluorescence intensity as the control and the siFPRL1 group (Fig. 3b). Finally, we tested the secretion of TGF-β1 by ELISA, which revealed that LXA4 could also suppress the secretion of TGF-β1. However, the knock down of FPRL1 eliminated the suppressive function of LXA4 (Fig. 3c).

### LXA4 reverses the mesenchymal phenotypes of pancreatic cancer cells via the inhibition of autocrine TGF-β1 signaling

**Fig. 4:**
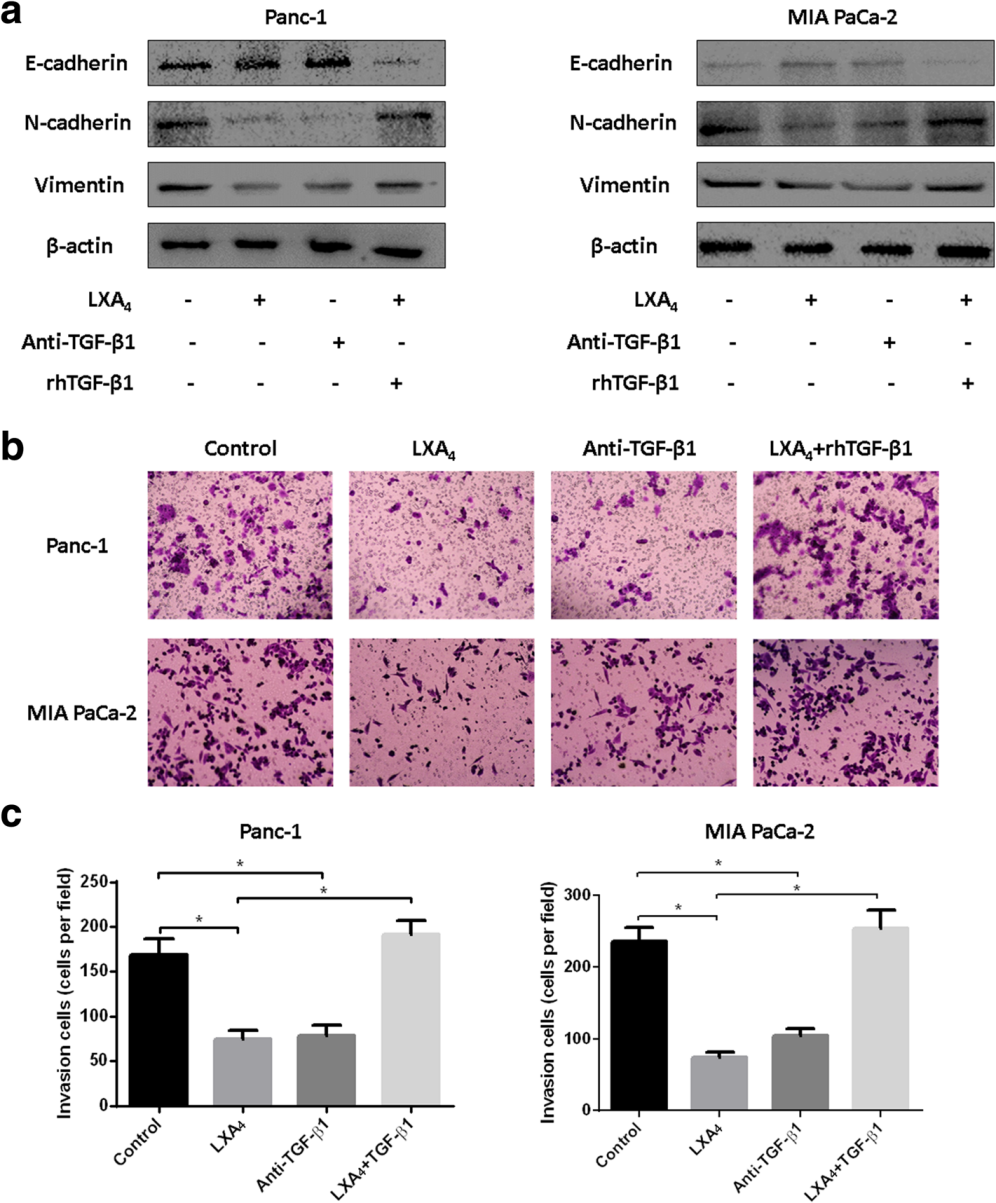
LXA4 reverses mesenchymal phenotypes of pancreatic cancer cells by inhibiting TGF-β1 autocrine. **a** Panc-1 and MIA PaCa-2 cells were treated with vehicle, LXA4 (400 nM), anti-TGF-β1 neutralized antibody (1 μg/mL), or combined treated with LXA4 and recombinant human TGF-β1 (rhTGF-β1, 5 ng/mL). Then western blotting was used to determine the expression of TGF-β1. **b** and **c** The former cells were seeded into matrigel-coated chambers to perform transwell invasion assays and the numbers of passed cells were counted in 10 random fields under 200× magnification at 0th and 24th hour. *, *P* < 0.05. All data were obtained from at least three independent experiments

TGF-β1 is a type of secreted protein that activates the TGF-β receptor, which influences cellular phenotypes [19]. To explore whether autocrine TGF-β1 signaling plays a role in the mechanism by which LXA4 suppresses the mesenchymal phenotypes in pancreatic cancer cells, anti-TGF-β1 neutralizing antibody and exogenous rhTGF-β1 were used to modulate the concentration of TGF-β1 in the cell culture media. When an anti-TGF-β1 neutralizing antibody (1 μg/mL) was added, E-cadherin expression was elevated while N-cadherin and Vimentin expression were decreased, which was similar to what was observed with LXA4 administration. Interestingly, when rhTGF-β1 (5 ng/mL) was administered after LXA4 treatment, the expression of EMT-associated markers was reversed (Fig. 4a). These data demonstrated that LXA4 attenuated mesenchymal phenotypes via the inhibition of autocrine TGF-β1 signaling. Then, Transwell invasion assay was performed to assess the invasive capacity of both Panc-1 and MIA PaCa-2 cells. Similarly, an anti-TGF-β1 antibody and LXA4 significantly decreased the number of passaged cells (*P* < 0.05), but rhTGF-β1 reversed the effect of LXA4 (*P* < 0.05) (Fig. 4b, c).

### The stable analog of LXA4, BML-111, suppresses liver metastasis of pancreatic cancer in vivo

**Fig. 5:**
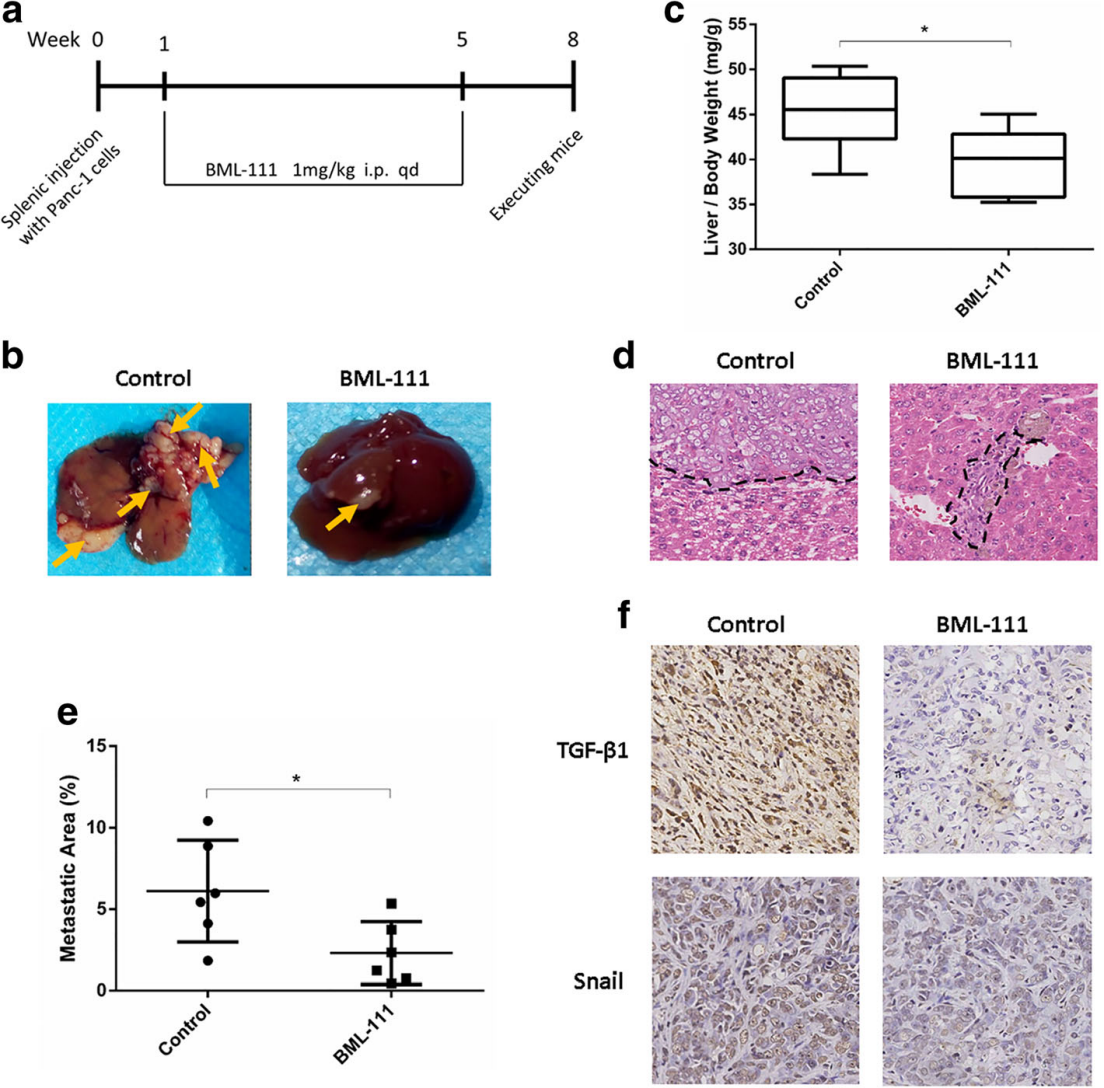
LXA4 stable analog BML-111 suppresses liver metastasis of pancreatic cancer in vivo*.* **a** The liver metastatic model of BALB/c nude mice was established and treated with vehicle (methanol) or BML-111 (1 mg/kg) as interpreted in this figure (*n* = 6). **b** At the end of eighth week, the livers were obtained and the metastatic lesions (yellow arrow) were observed. **c** Liver indexes (liver weight/body weight, mg/g) were calculated. **d** and **e** Each liver was randomly sliced into 6 parts and made into paraffin-embedded sections. Each section was observed under microscope to calculate relative area of metastatic lesions. The final result was the average metastatic area in 6 parts. **f** Primary tumors in the spleen were stained immunohistochemically for TGF-β1 and Snail. *, *P* < 0.05

Our data revealed that LXA4 could attenuate the invasiveness of pancreatic cancer in vitro. Whether LXA4 could inhibit metastasis in vivo remained to be investigated. Thus, we established a liver metastasis model by injecting Panc-1 cells into the subsplenic membranes of mice. One week after the model was established, mice were treated with vehicle or BML-111, which is a stable analog of LXA4 and an FPRL1 agonist. The drug was administered for five weeks, and all of the mice were sacrificed at the end of the eighth week (Fig. 5a). We found that the mice that were treated with BML-111 exhibited fewer metastatic lesions and lower relative liver weights compared to mice in the control group (Fig. 5b, c). By microscopy, larger areas of metastatic lesions in the liver were found in mice treated with vehicle, whereas in mice treated with BML-111, the lesions were small and most of them were located near blood vessels (Fig. 5d, e). To confirm the mechanisms that were explored in vitro, we determined the expression of TGF-β1 and that of a key EMT-promoting transcription factor, Snail, by immunohistochemistry. We found that both TGF-β1 and Snail were down-regulated by BML-111 (Fig. 5f), which suggested that similar mechanisms occurred in vivo.

### Low Lipoxin effect score (LES) is correlated with aggressive metastatic potential

**Fig. 6:**
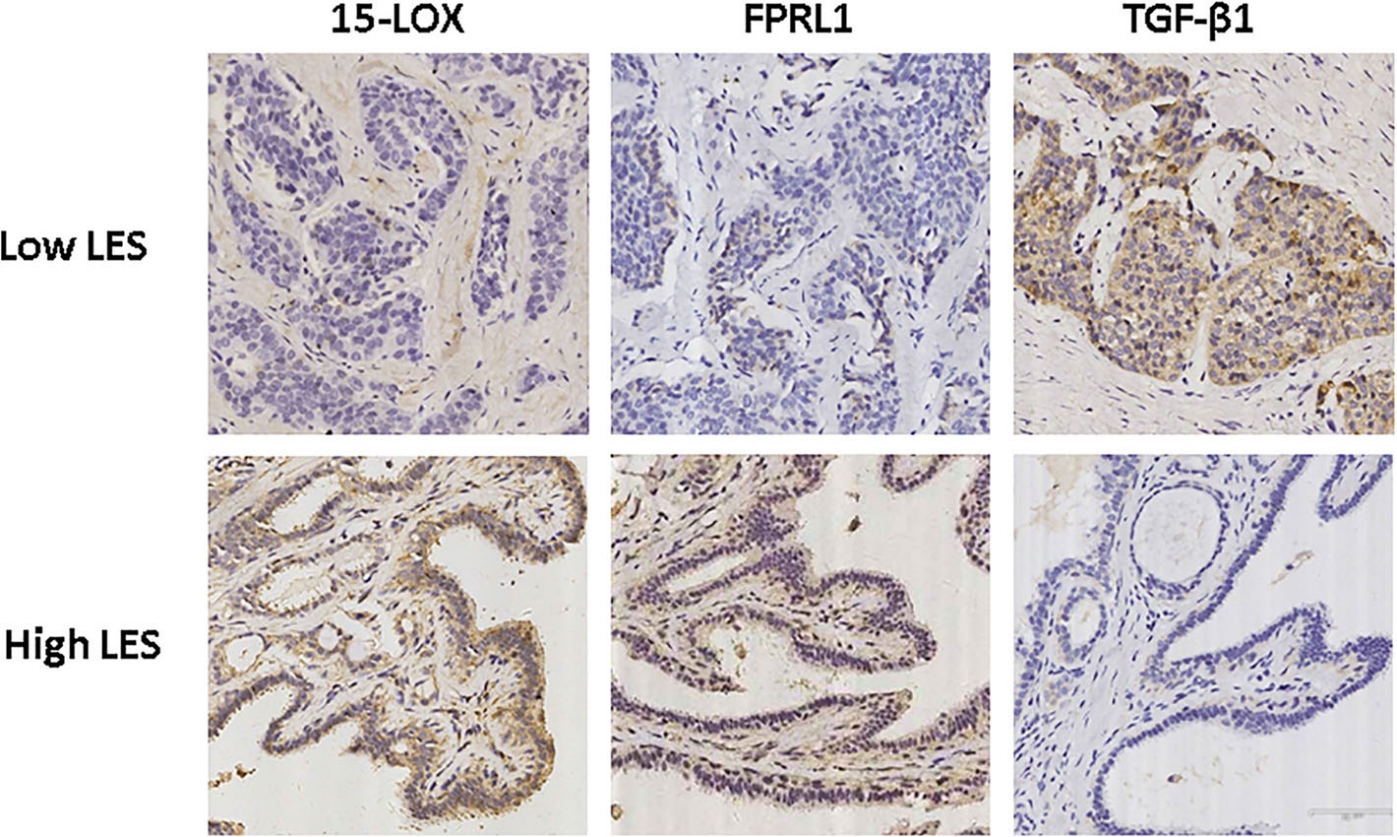
Low Lipoxins effect score (LES) is correlated with aggressive metastatic potential. Pancreatic cancer tissues embedded with paraffin from 66 patients with complete records was performed immunohistochemical staining for 15-LOX, FPRL1 and TGF-β1. Based on the histological scores of 15-LOX and FPRL1, Lipoxins effect score (LES) could be calculated

LXA4 is an endogenous lipid molecule that exerts its biological activity via FPRL1. We wondered whether the concentration of LXA4 in human pancreatic cancer tissues correlates with the metastatic potential. However, the concentration of LXA4 in tissues is extremely low (~0.1 ng/mL) [20] and is therefore difficult to extract. Above all, it is difficult to evaluate the biological activity of LXA4 solely by its concentration while the expression of its receptor FPRL1 is ignored. To solve this problem, we defined the “Lipoxin effect score” (LES) as the product of the immunohistochemistry scores of both 15-LOX and FPRL1. Because immunohistochemistry scores range from 0 to 3, the LES can be a number in the set (0, 1, 2, 3, 4, 6, 9). We also define a low LES (LES < 4) and a high LES (LES ≥ 4). Then, we analyzed the correlation between the LES and the clinical-pathological features of pancreatic cancer (Table 1). We found that the LES correlated with histological grade, primary tumor (T), lymph node metastasis (N), and distant metastasis (M). Specifically, a low LES tended to correlate with lymph node and distant metastasis, which confirmed our previous results in vitro and in vivo. In addition, immunohistochemical staining revealed that patients with a low LES tended to express more TGF-β1 and vice versa (Fig. 6). Statistical analysis revealed that the LES was also correlated with TGF-β1 expression (Table 2), which further confirmed that LXA4 attenuated cell invasion and metastasis via the inhibition of TGF-β1 expression.

**Table 1:** Correlation between Lipoxins Effect Score (LES) and Clinical Pathological Features in Pancreatic Cancer

	Sample (n)	Lipoxins Effect Score (LES)		
		Low LES (%)	High LES (%)	*P* Value^∆^
Gender				0.295
Male	39	21 (53.8)	18 (46.2)	
Female	27	11 (40.7)	16 (59.3)	
Age				0.622
< 56.50†	33	15 (45.5)	18 (54.5)	
≥ 56.50	33	17 (51.5)	16 (48.5)	
Types				1.000
Ductal Adenocarcinoma	62	30 (48.4)	32 (51.6)	
Other Types	4	2 (50.0)	2 (50.0)	
Histological Grade				0.029*
1	20	6 (30.0)	14 (70.0)	
2	23	10 (43.5)	13 (56.5)	
3	23	16 (69.6)	7 (30.4)	
pT Status				0.030*
T1	3	0 (0.0)	3 (100.0)	
T2	21	7 (33.3)	14 (66.7)	
T3	39	22 (56.4)	17 (43.6)	
T4	3	3 (100)	0 (0.0)	
pN Status				0.008*
N0	54	22 (40.7)	32 (59.3)	
N1	12	10 (83.3)	2 (16.7)	
pM Status				0.026*
M0	60	26 (43.3)	34 (56.7)	
M1	6	6 (100.0)	0 (0.0)	
TNM Stage (AJCC)				0.002*
I	19	4 (21.1)	15 (78.9)	
II	32	15 (46.9)	17 (53.1)	
III	9	7 (77.8)	2 (22.2)	
IV	6	6 (100.0)	0 (0.0)	

Δ χ^2^ test; † Median age; * Significant difference, *P* < 0.05

**Table 2:** Correlation between Lipoxins Effect Score (LES) and TGF-β1 Expression

	Lipoxins Effect Score (LES)		
	Low LES	High LES	*P* Value ^Δ^
TGF-β1 Expression			0.036*
Low	8	17	
High	24	17	

Δ χ^2^ test; * Significant difference, *P* < 0.05

## Discussion

Traditionally, LXA4 has been regarded as a small anti-inflammatory lipid molecule. Recently, it has also been reported to be a novel anti-cancer agent in hepatocellular carcinoma. On the one hand, LXA4 inhibited proliferation, promoted apoptosis, and suppressed EMT and invasion via the inhibition of the nuclear factor–κB (NF-κB) pathway [11]; on the other hand, distant metastasis was attenuated by LXA4 due to suppression of angiogenesis [12]. Pancreatic cancer tends to exhibit aggressive invasion and metastatic potential, which is the main reason why patients with this disease usually have a very poor prognosis [1]. Here, we showed that LXA4 could also inhibit the migration and invasiveness of pancreatic cancer.

Aggressive cancer cells tend to exhibit obvious mesenchymal phenotypes, including loss of intercellular connections and acquisition of flexible plasticity, which facilitate local invasion and distant metastasis [21]. According to previous studies, both hyperglycemia and hypoxia share the same mechanism whereby mesenchymal phenotypes are enhanced through EMT to promote invasion and metastasis in pancreatic cancer [22, 23]. In the present study, we revealed that LXA4 suppressed the expression of mesenchymal markers but up-regulated the expression of epithelial markers in a concentration-dependent manner. This result demonstrated that LXA4 might attenuate invasion and metastasis via the inhibition of EMT. Recent studies [24, 25] have shown that cancer cells attain a mesenchymal phenotype via EMT. Cancer cells also typically express molecular markers similar to those expressed in cancer stem cells (CSCs), such as CXC receptor 4 (CXCR4). Pancreatic CSCs distinguished by CXCR4^+^ expression have a strong capacity to invade adjacent tissues and disseminate to distant organs [26]. In addition, the stromal cell-derived factor (SDF)-1/CXCR4 axis also enhanced EMT [3, 4] and perineural invasion [27]. Therefore, LXA4 may also decrease the number of CXCR4^+^ CSCs. In addition to the suppression of mesenchymal phenotypes, LXA4 can also down-regulate matrix metalloproteinases (MMPs), such as MMP-9 and MMP-2, even under hypoxic conditions [13].

LXA4 exerts its biological activity through binding to its receptor, FPRL1. In the present study, we knocked down FPRL1 expression by siRNA, which reversed LXA4-suppessed mesenchymal phenotypes; this suggests that FPRL1 mediates the function of LXA4. Actually, LXA4 is not the only ligand that binds to FPRL1. Other lipid mediators such as Resolvin D1 (RvD1), which is metabolized from docosahexaenoic acid, can also bind to this receptor [28]. Interestingly, RvD1 has also been reported to inhibit EMT through FPRL1 in lung cancer [29]. BML-111, which was used in this study, is another FPRL1 agonist that attenuates liver metastasis. These data imply that FPRL1 may be a novel target for the prevention of invasion and metastasis in pancreatic and other cancers. However, one study has demonstrated that LXA4 may not activate FPRL1 as efficiently as was once thought [30], which implies that LXA4 may act in an FPRL1-independent manner. However, in the present experiment, we confirmed the key role that FPRL1 plays in the attenuation of mesenchymal phenotypes by LXA4.

TGF-β1 is a canonical factor that facilitates the maintenance of mesenchymal phenotypes and the metastatic capacity of cancer cells [17]. Here, we found that LXA4 dramatically down-regulated the expression of TGF-β1. We used an anti-TGF-β1 neutralizing antibody and exogenous rhTGF-β1 to confirm that LXA4 inhibited autocrine TGF-β1 signaling and further influenced cellular phenotypes. Moreover, this phenomenon was also directly confirmed in a liver metastasis model in nude mice and was indirectly confirmed in clinical-pathological sample analyses. In addition to the promotion of EMT and metastasis, TGF-β1 is also a key enhancer of tissue fibrosis [31, 32]. Desmoplasia, which is another characteristic of pancreatic cancer, occurs as a result of excessive extracellular matrix components produced by activated pancreatic stellate cells (PSCs) [33]. Although the role of PSCs in pancreatic cancer progression is still controversial [34], we have previously shown that PSC activation and desmoplasia lead to poor outcomes of pancreatic cancer patients [35–37]. Various studies have demonstrated the fibrosis-suppressive function of LXA4 in the lungs [38], kidneys [39], liver [40] and epidermis [41]. Therefore, we can infer that LXA4 may attenuate desmoplasia via the inhibition of TGF-β1 secretion, which may retard the progression of pancreatic cancer. However, further studies are still required to address the role of LXA4 in the desmoplasia of pancreatic cancer.

In the last part of the study, we defined the LES to evaluate the actual biological effects of LXA4 in vivo. This scoring system has several advantages compared with the direct determination of the concentration of LXA4 in pancreatic cancer tissues. The LES avoids complicated extraction methods such as liquid chromatography/tandem mass spectrometry. Only the use of immunohistochemistry can accomplish this evaluation. More importantly, the LES links the production and biological activities of LXA4, which seems to reflect its actual effects in tissues more comprehensively. Additionally, a similar method may be applied to the clinical diagnosis of cancer in the future.

## Conclusions

In summary, our results revealed that a low LES was correlated with aggressive invasion and metastatic potential in pancretic tissues from patients with pancreatic cancer. By binding to its receptor FPRL1, LXA4 could attenuate the invasiveness of pancreatic cancer cells through the suppression of the mesenchymal phenotypes. In addition, our results suggested that the effect of LXA4 in pancreatic cancer was in part mediated by inhibition of the TGF-β1 autocrine signaling. This study also implied that the development of 15-LOX stimulators or FPRL1 agonists may be a new strategy to block local invasion and distant metastasis in pancreatic cancer.

## Abbreviations

15-LOX: 15-lipoxygenase
CSCs: Cancer stem cells
CXCR4: CXC receptor 4
DMEM: Dulbecco’s Modified Eagle’s Medium
EMT: Epithelial-mesenchymal transition
FBS: Fetal bovine serum
FPRL1: Formyl peptide receptor like-1
LES: Lipoxin effect score
LXA4: Lipoxin A4
MMPs: Matrix metalloproteinases
NF-κB: Nuclear factor–κB
PSCs: Pancreatic stellate cells
SDF-1: Stromal cell-derived factor-1
TGF-β1: Transforming growth factor-β1
